# Pelvic floor muscle training with biofeedback or feedback from a physiotherapist for urinary and anal incontinence after childbirth - a systematic review

**DOI:** 10.1186/s12905-023-02765-7

**Published:** 2023-11-18

**Authors:** Amanda Höder, Josefin Stenbeck, Mia Fernando, Elvira Lange

**Affiliations:** 1 Unit of Physiotherapy, Department of Health and Rehabilitation, Institute of Neuroscience and Physiology, Sahlgrenska Academy, University of Gothenburg, Gothenburg, Sweden; 2 Meja Women’s health, Stockholm, Sweden; 3 Unit of Physiotherapy, Department of Health and Rehabilitation, Institute of Neuroscience and Physiology, Department of General Practice/Family Medicine, School of Public Health and Community Medicine, Institute of Medicine, Sahlgrenska Academy, Sweden. Research, Education, Development and Innovation, Primary Health Care, Region Västra Götaland, Gothenburg, Sweden

**Keywords:** Physical therapy, Postpartum, Grading of evidence

## Abstract

**Background:**

Childbirth is one of the biggest risk factors for incontinence. Urinary and anal incontinence can cause pain and social limitations that affect social life, cohabitation, and work. There is currently no up-to-date literature study on the effect of pelvic floor muscle training with feedback from a physiotherapist, which involves verbal instructions based on vaginal and anal digital palpation, compared to treatment without feedback (e.g., recommendations for pelvic floor muscle training).

**Aim:**

The objective of this systematic review was to examine the scientific evidence regarding the impact of pelvic floor muscle training (PFMT) with feedback from a physiotherapist and/or biofeedback on urinary and anal incontinence in women during the first six months following vaginal delivery, compared to treatment without feedback.

**Methods:**

The literature search was conducted in the databases PubMed, Cochrane, and CINAHL. In addition, a manual search was conducted. The search terms consisted of MeSH terms and synonyms in the respective search block including population, intervention, and study design, as well as the terms pelvic floor and postpartum. An evaluation of each included study was conducted for methodological quality, evidence value, and clinical relevance.

**Results:**

Eight studies were included, three of which showed a significant difference between groups, in favor of the intervention group that received pelvic floor muscle training with feedback from a physiotherapist and/or biofeedback. Due to the varying results and insufficient quality for the majority of the studies, the scientific basis was considered insufficient.

**Conclusion:**

The scientific evidence for pelvic floor muscle training with feedback from a physiotherapist or biofeedback on postpartum urinary and anal incontinence compared to treatment without feedback is considered insufficient. Further research on the subject is needed. *The study is registered in PROSPERO CRD42022361296.*

**Supplementary Information:**

The online version contains supplementary material available at 10.1186/s12905-023-02765-7.

## Background

Urinary incontinence (UI) is defined as the involuntary leakage of urine through the urethra [1]. Anal incontinence (AI) is defined as the involuntary passage of gas or stool (solid or liquid) through the anal canal [2, 3]. Vaginal delivery is associated with a higher risk for UI, and operative vaginal delivery is associated with AI [4, 5]. The reported prevalence of UI in the postpartum period ranges from 3 to 40% [6–8]. The prevalence of AI following vaginal delivery is between 5 and 26% [2, 3]. Although there is a natural history of recovery of pelvic floor structures during postpartum, UI does not always resolve due to this recovery [9].

Moreover, UI and AI are associated with significant reductions in health-related quality of life [10–13]. Women who experience a new onset of AI after childbirth report persistently negative quality of life as long as two years after delivery [14]. Patients in the general population with UI may have higher rates of depression and anxiety than individuals without UI [15]. UI is also associated with postpartum depression in the first six months after childbirth [16].

Urinary and anal incontinence is often assessed through self-assessment questionnaires [17, 18] containing questions about symptoms, frequency, and when symptoms occur. In addition, self-assessment questionnaires usually include questions about self-reported health and quality of life, as urinary incontinence is a barrier to women’s participation in sports and fitness activities and therefore may be a threat to women’s health, self-esteem, and well-being [19, 20]. There is some evidence to suggest that women with UI and AI may benefit from pelvic floor muscle training (PFMT) and other rehabilitative care, commonly referred to as pelvic floor physiotherapy, during the postpartum period [21, 22].

PFMT has been shown to reduce UI and may reduce AI [23, 24]. It is recommended as the first line treatment for all women with UI and/or AI [25]. Feedback from a physiotherapist or biofeedback may provide benefits in addition to PFMT in women with UI [26]. Biofeedback in combination with PFMT has been shown to reduce UI [26] and to potentially reduce AI [23] in adults. However, there is uncertainty about the effect of PFMT as a treatment for UI in postpartum women [21].

Feedback is the sensory information that becomes accessible following an individual’s conducted activity [27]. Feedback from a physiotherapist, in PFMT consist of verbal instructions based on vaginal and anal digital palpation, to ensure a correct contraction [28]. The main mechanism of action for feedback in training is an increased adherence to the training program [29]. Biofeedback entails utilising an external sensor to provide insight into bodily processes, typically with the aim of modifying the measured quality [30] and could be used both superficially and intravaginally and/or intra-analy [23]. The technique aims to make the patient aware of a usually unconscious bodily function, where the mechanisms of action behind reduced UI and AI could be increased rectal sensitivity, increased strength, and coordination [23].

In recent years, various systematic reviews have been published in the field. Zhu et al. [31] investigated the effect of PFMT with biofeedback and/or electrical stimulation in women after childbirth. The results showed that PFMT and electrical stimulation with or without biofeedback were more effective than PFMT alone [31]. Mazur et al. [23] investigated the efficacy of preventive and therapeutic physiotherapy in women with UI and AI after childbirth. The results showed that physiotherapeutic interventions, including PFMT, biofeedback, and/or electrical stimulation can be effective in reducing incontinence symptoms [23].

To the authors’ knowledge, there is no systematic review that specifically reports the effect of PFMT with feedback from a physiotherapist or biofeedback in UI and AI after childbirth. Previously mentioned studies have not focused on only feedback and/or biofeedback [17, 23]. Additional studies have been published since these reviews were performed, and there is a need for an overview that compiles the current evidence in the field.

The objective of this systematic review was to examine the scientific evidence from 2012 to 2022 regarding the impact of pelvic floor muscle training (PFMT) with feedback from a physiotherapist and/or biofeedback on UI and AI in women during the first six months following vaginal delivery, compared to treatment without feedback.

## Method

A systematic literature search was carried out based on previously established selection criteria to achieve the purpose of this systematic review (see Table 1). The Cochrane handbook [32] and PRISMA guidelines [33] were followed. The inclusion criteria were expanded after the protocol was registered due to the low number of relevant randomised controlled trials.

**Table 1:** Inclusion and exclusion criteria

Inclusion criteria	Exclusion criteria
· Studies examining women over 18 who have had a vaginal birth. · Studies with pelvic floor muscle training programs with biofeedback or feedback, including digital palpation, from a physiotherapist. · Studies where the control intervention consisted of other treatment that does not consist of feedback of any type, e.g. recommendations on pelvic floor training. · Studies that reported the effect of exercise on problems with urinary and/or anal incontinence with self-assessment questionnaires. · Study design is a randomised controlled trial or controlled clinical trial. · Studies must be approved by an ethics review board or comply with the Declaration of Helsinki. · The literature must be in English.	· Studies examining women who had urinary and/or anal incontinence before pregnancy. · Studies that introduced training more than 6 months after delivery. · Studies that also investigated the effect of exercise for prevention of incontinence. · Studies published earlier than 2012.

The systematic literature search was conducted independently by AH and JS on 30 September 2022 in the databases PubMed, Cochrane, and CINAHL. To ensure the inclusion of recent studies, the time limit 2012–2022 was used. In the search, five search blocks were used with keywords from MeSH terms and synonyms for these. Search block #1 included the population. Search block #2 consisted of pelvic floor terms. Search block #3 included the intervention. Search block #4 consisted of the term postpartum, and search block #5 included a description of the study design. The same search blocks were used in all databases (see Table 2). A complementary search to present date, was performed in October 2023, where the term ‘physical therapy’ was added, with no additional findings included. For the full search strategy, see Additional file 1.

**Table 2:** Reporting of the number of hits per search block and total hits

Search block	PubMed	Cochrane	CINAHL
#1	51,880	11,016	4,479
#2	9,705	3,778	1,101
#3	2,669,175	199,031	125,079
#4	53,944	10,218	6,066
#5	1,431,020	1,282,034	102,239
#1 + #2 + #3 + #4 + #5	102	220	7

The selection process began with AH and JS individually reading and reviewing all titles. The titles that were consistent with the purpose of the study were retained. The authors individually assessed the titles, and differences were discussed until a consensus was reached. After agreement on relevant titles, duplicates were excluded. In the next step, the authors reviewed abstracts individually based on inclusion and exclusion criteria. AH and JS discussed the assessment until a consensus was reached. In case of uncertainty, the title was kept to the next step. The following step included individual readings of the full texts, where the studies were reviewed based on the inclusion and exclusion criteria. The assessment was then discussed until a consensus was reached (including EL), and the remaining studies were included in the systematic review.

A manual search was performed in the reference lists of the included studies and in relevant systematic reviews. Review of the reference lists was done using a process similar to the previous selection process in the database search. In each step the authors assessed the studies individually and thereafter discussed until a consensus was reached.

### Methodological quality

The PEDro scale was used to assess methodological quality [34]. It aims to assess the methodological quality of clinical studies based on eleven criteria [35]. The first criterion consists of external validity. Criteria 2–9 consist of internal validity and criteria 10–11 of statistical reporting. The maximum score was 10 since external validity is not included in the overall assessment. The assessment was performed individually by AH, and JS and a single score was agreed up on. Studies with a total score of < 4 were considered to have a low methodological quality [35]. Total points of 4–5 were considered medium quality, and studies with 6–8 points were considered high methodological quality. Studies with 9–10 points were considered to have excellent quality [35].

### Probative value

After assessment of methodological quality, the studies were reviewed for probative value [36]. The probative value of the studies was graded as high, medium, or low. When assessing the probative value of studies, no absolute limits were applied [36]. The grading was based on the methodological quality of the study, as well as the appropriateness of the study design and the size of the study. The authors considered aspects such as randomisation, the similarity of the groups at baseline, and the number of dropouts when assessing the appropriateness of the study design. The size of the study was considered adequate if a power calculation or sample size analysis had been performed and a sufficient number of participants were included.

### Assessment of clinical relevance

The clinical relevance of the results was assessed based on five questions [37] (see Table 3). The questions were answered yes, no, or unclear [37]. Questions 1–3 assess applicability, and questions 4–5 assess clinical relevance [38]. To assess descriptions of interventions, particular descriptions of specific exercises where sought. As this review was not restricted to specific outcome measures, effect sizes were calculated manually, and effect size cut-offs were used according to Cohen’s d [37]. At a Cohen’s d below 0.5, the effect was considered to be small; at 0.5 < 0.8, the effect was considered to be moderate; and at Cohen’s d ≥ 0.8, the effect was considered to be large [37]. The effect size was calculated manually with a calculator (Effect size calculators, University of Colorado, US) according to the formula Cohen’s d = M 1 - M 2 / s pooled where s pooled = √[ (s 1 2 + s 2 2 ) / 2]. At least moderate effect was required for the study to be considered clinically relevant in this systematic review. The studies were considered clinically relevant with five yes replies.

**Table 3:** Questions for assessment of clinical relevance [37]

Area:	Question:
1. Detailed description of participant	Are the patients described in detail so that you can decide whether they are comparable to those that you see in your practice?
2. Detailed description of interventions	Are the interventions and treatment settings described well enough so that you can provide the se for your patients?
3. Use and report of clinically relevant outcome measures	Were all clinically relevant outcomes measured and reported?
4. Clinically important effect size	Is the size of the effect clinically important?*
5. Benefits overcome potential harms	Are the likely treatment benefits worth the potential harms?

*Effect size cutoffs: small effect = Cohen’s d below 0.5, moderate effect = Cohen’s d from 0.5 < 0.8, large effect = Cohen’s d ≥ 0.8

### Grading of evidence

Grading of evidence was based on Britton’s model [36]. The level of evidence was graded as strong, moderately strong, limited, or insufficient (see Table 4). When assessing the level of evidence, the authors accounted for the probative value and consensus in the results, as well as the clinical relevance of the studies. In the event of contradictory results in the studies, the level of evidence was lowered.

**Table 4:** Grading of evidence [36]

1: Strong scientific basis	At least two studies with a high level of evidence or a good systematic review.
2: Moderately strong scientific basis	One study with a high level of evidence plus at least two with a medium level of evidence.
3: Limited scientific basis	At least two studies with a medium level of evidence.
4: Insufficient scientific basis	Everything below the abovementioned criteria. Studies with low evidence value.

* In the event of contradictory results in the studies, the level of evidence is lowered.

## Results

**Fig. 1:**
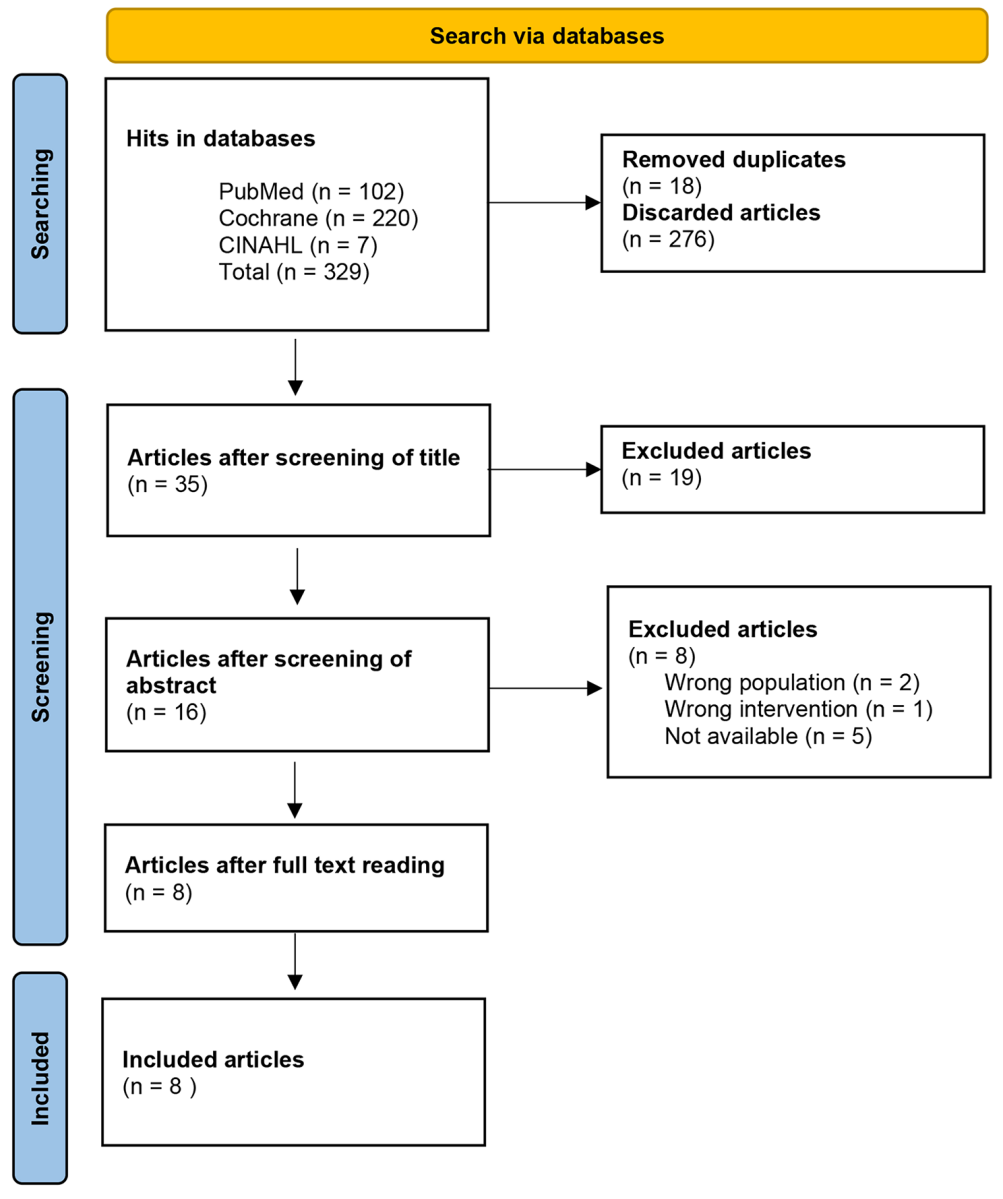
Flow chart of the selection process reported according to PRISMA [33]

A total of 329 records were selected in the database searches. After removing irrelevant titles and duplicates, 35 abstracts were reviewed. Of these, 16 studies were selected for full-text reading (List of articles not included after full-text reading are presented in Additional file 2), resulting in eight studies [39–46] that were included in the systematic review (see Fig. 1). The manual search was carried out in reference lists of the included studies [39–46] and from seven systematic reviews [21, 23, 25, 31, 47–49]. The manual search did not lead to the inclusion of any additional studies.

### Characteristics of included studies

A total of 765 women who underwent a vaginal delivery participated in the included studies (see Table 5 for details). There were five studies that included women with perineal lacerations and levator ani injuries [40, 42, 43, 45, 46]. The remaining studies included women with incontinence diagnosed based on self-assessment questionnaires [39, 40, 42]. Of the included studies, three studies had the self-assessment of both UI and AI as outcome measures [41, 44, 45]. Three studies studied only UI [39, 40, 46] and two studies only AI [41, 43]. The mean age of the women in the studies was 28.5 (± 4.8) − 32.1 (± 4.9). Six of eight studies only included primiparous women [39, 40, 42–44, 46], in one study the mean number of pregnancy was 1.4 [41] and in one it was 1.1 [45].

Of the eight studies included, two studies reported using biofeedback devices only [42, 43], two studies reported using a combination of biofeedback devices and feedback from a physiotherapist [44, 46] while four reported participants receiving feedback from a physiotherapist [39–41, 45]. Initially, all participants, except those included in the study by Peirce et al. [43], were given instructions to perform correct pelvic floor contraction. Three studies reported using biofeedback in the daily training at home [42, 43, 46], and one study used biofeedback during clinical training sessions [44]. The studies used different devices, Myotrac Infiniti Vaginal Sensor [42], CombiStim XP Neurotech® [43], NeuroTrack Simplex [44] and Enraf-Nonius Myomed134 [46], which all provide visual feedback on muscle contractions with electromyography (EMG) biofeedback. In the other four studies, participants received feedback from a physiotherapist [39–41, 45]. In all intervention groups, participants received a home exercise program with PFMT. Participants in two of the studies used biofeedback at home in their daily training [43, 46]. The duration of the interventions ranged from 6 weeks to 6 months and differed slightly between studies. The intervention included follow-up with the participants, but the timing varied from weekly to every six weeks. For a more detailed description of the interventions used in the studies, see Table 5.

In five of the studies, participants in the control group received instructions to perform a correct pelvic floor contraction [39–41, 44, 46]. Of these, three studies provided the control groups with recommendations to continue carrying out PFMT [39, 41, 46]. In one of the included studies, the control groups only received recommendations about PFMT [43]. The participants in the control groups of Oakley et al. [42] and Von Bargen et al. [45] received standard care, which was not specified.

## Results of included studies

Three of eight studies reported a significant difference between the groups, with participants in the intervention groups showing reduced incontinence symptoms [41, 44, 45]. All three studies evaluated AI symptoms [41, 44, 45], where two reported a significant difference between the groups [41, 45]. Two studies evaluated UI symptoms and both reported a significant difference between the groups [44, 45]. The included studies used different self-reported questionnaires to evaluate outcomes ( Table 5). In all questionnaires, a lower total score indicated less symptoms of incontinence, and all questionnaires were reported to be validated. One of the included studies compared standard care with an individualised daily home PFMT program and weekly verbal and tactile feedback on correct pelvic floor contraction from a physiotherapist [45]. After three months, a significant difference was observed between the groups in terms of urinary and AI symptoms [45]. The second study examined an individualised and progressive daily home exercise program delivered by a physiotherapist that included advice on PFMT. AI symptoms were evaluated after six months, and a significant difference between the groups was observed [41]. The third study examined an individualised daily home exercise program and 12 training sessions with biofeedback compared to PFMT instructions [44]. After six months, there was a significant between-group difference in symptoms of UI, but no difference in AI symptoms. After 12 months, no significant difference was reported between the groups [44]. In five of the studies, no significant between-group differences were reported [39, 40, 42, 43, 46]. Three of the included articles reported a significant difference in the reduction of incontinence symptoms within both the intervention group and the control group after three [42] and six months [39, 41], respectively.

### Methodological quality

Methodological quality was assessed with the PEDro scale (see Table 5). Three studies were considered to have high methodological quality [40, 44, 45], four were considered to have medium methodological quality [39, 41–43], and one was judged to have low methodological quality [46]. Seven studies performed a randomisation [30–42, 44–46], and six of these were concealed [39, 40, 42–45]. Five studies reported similarities between the groups at baseline [39, 41, 44–46]. None of the studies had blinded participants, therapists, or assessors. Four studies had more than 15% participant dropout [39, 41, 45, 46]. Three studies carried out an intention-to-treat analysis [40, 41, 45]. For a full presentation of the assessment of methodological quality, see Additional file 3.

**Table 5:** Reporting of included studies. Accounting of variables, see respective results column.

Author, year, study design, country	Population and sample *Mean (SD)*	Intervention	Control group	Evaluation and outcome measures	Results *Types of Measures of Central Tendency and Dispersion are specified below*
Ahlund et al. 2013 [39] RCT Sweden	Women with UI after childbirth. Mean age: 33 (3.6) Mean number of pregnancies: primiparous women only Number randomized: n = 98 Intervention: n = 49 Control: n = 49	Start: 10–16 weeks pp. Duration: 6 months. PMFT + feedback from physiotherapist Instructions through palpation, verbal, and written instructions on correct pelvic floor contraction, as well as a 15-minute lesson on anatomy and function of the pelvic floor muscles. A daily training program and continuous follow-up every six weeks with continued tactile guidance and feedback.	Instructions through palpation, verbal, and written instructions on proper pelvic floor contraction. Brief written information about pelvic floor anatomy and recommendations for pelvic floor training.	Evaluation: 6 months. Bristol Female Lower Urinary Tract Symptoms (ICIQ FLUTS) Secondary outcome measure.	No significant difference between groups. Significant difference within both groups. Intervention group: UI, *Median (range)* ICIQ FLUTS: Baseline: 7 (1–16) ICIQ FLUTS: Evaluation: 4 (9–15) Control group: UI, *Median (range)* ICIQ FLUTS: Baseline: 7 (2–16) ICIQ FLUTS: Evaluation: 4 (0–12)
Hilde et al. 2013 [40] RCT Norway	Women with and without UI after delivery, with or without levator injury. Mean age: 29.8 (4.1) Mean number of pregnancies: primiparous women only Number randomized: n = 175 Intervention: n = 77 Control: n = 88	Start: 6 weeks pp. Duration: 4 months. PMFT + feedback from physiotherapist Instructions through palpation, and verbal instructions on proper pelvic floor contraction. Supervised pelvic floor training with a physiotherapist once a week and daily pelvic floor training. Daily recorded exercise diary.	Instructions through palpation, and verbal instructions on proper pelvic floor contraction.	Evaluation: 6 months The International Consultation on Incontinence Questionnaire–Urinary Incontinence Short Form (ICIQ-UI SF) Primary outcome measure.	No significant difference between groups. Intervention group: UI, *n (%)* ICIQ-UI SF: Baseline: 34 (39.1%) ICIQ-UI SF: Evaluation: 30 (34.5%) Control group: UI, *n (%)* ICIQ-UI SF: Baseline: 44 (50.0%) ICIQ-UI SF: Evaluation: 34 (38.6%)
Johannessen et al. 2017 [41] RCT Norway	Women with AI after childbirth. Mean age: 30.1 (4.1) Mean number of pregnancies: 1.4 Number randomized: n = 109 Intervention: n = 54 Control: n = 55	Start: Not reported Duration: 6 months. PMFT + feedback from physiotherapist Instructions on correct pelvic floor contraction. Individualized and progressive daily home training program by physiotherapist with 4–6 follow-ups. Training diary.	Instructions on correct pelvic floor contraction. Advice on pelvic floor exercises but no encouragement.	Evaluation: 6 months. St. Mark’s scores. Primary outcome measure.	Significant difference between groups. (AI: p = 0.04). Significant difference within both groups. Intervention group: AI, *Mean (SD)* St. Mark’s scores: Baseline: 5.4 (3.6) St. Mark’s scores: Evaluation: 3.3 (3.5) Control group: AI, *Mean (SD)* St. Mark’s scores: Baseline: 5.0 (3.2) St. Mark’s scores: Evaluation: 4.2 (3.4)
Oakley et al. 2016 [42] RCT USA	Women with grade 3–4 puerperal hernia. Mean age: 29.8 (4.66) Mean number of pregnancies: primiparous women only Number randomized: n = 54 Intervention: n = 29 Control: n = 25	Start: 6 weeks pp. Duration: 3 months. PMFT + feedback from biofeedback Information about the function and anatomy of the pelvic floor. Pelvic floor training with biofeedback 60 minutes every two weeks. Progressive home training program by physiotherapist with follow-up.	Standard care, not specified.	Evaluation: 3 months. Urinary Distress Inventory, Short Form (UDI – 6) and the Fecal Incontinence Severity Index (FISI) Secondary outcome measures.	No significant difference between groups. Significant difference within both groups. Intervention group: UI, *Mean (SD)* UDI – 6: Baseline: 19.44 (27.78) UDI – 6: Evaluation: 0.0 (12.50) AI, *Mean (SD)* FISI: Baseline: 12.0 (25.0) FISI: Evaluation: 6.0 (20.5) Control group: UI, *Mean (SD)* UDI – 6: Baseline: 25.0 (40.27) UDI – 6: Evaluation: 11.11 (37.50) AI, *Mean (SD)* FISI: Baseline: 14.0 (18.0) FISI: Evaluation: 13.5 (22.25)
Peirce et al. 2013 [43] RCT Ireland	Women with grade 3 puerperal hernia. Mean age: Not stated Mean number of pregnancies: primiparous women only Number randomized: n = 120 Intervention: n = 30 Control: n = 90	Start: 24 hours pp. Duration: 3 months. PMFT + feedback from biofeedback Verbal and written information about technical equipment and training. Daily pelvic floor training with biofeedback.	Written information on daily pelvic floor training.	Evaluation: 3 months. Cleveland Clinic Continence Score Primary outcome measure.	No significant difference between groups. Intervention group: AI: Baseline: Not available. Evaluation: Not reported in text format, only indecipherable diagram Control group: AI: Baseline: Not available. Evaluation: Not reported in text format, only indecipherable diagram
Sigurdardottir et al. 2020 [44] RCT Iceland	Women with UI and AI after childbirth. Mean age: 28.5 (4.8) Mean number of pregnancies: primiparous women only Number randomized: n = 84 Intervention: n = 41 Control: n = 43	Start: 9 weeks pp. Duration: 3.7 months. PMFT + feedback from biofeedback and physiotherapist Instructions through palpation, and verbal instructions on proper pelvic floor contraction. 12 training sessions with biofeedback, 45–50 minutes, with a physiotherapist. Individualized and progressive daily home exercise program by physiotherapist. Training diary.	Instructions through palpation, and verbal instructions on proper pelvic floor contraction.	Evaluation: 6 and 12 months. Australian Pelvic Floor Questionnaire (APFQ) Primary outcome measure.	At 6 months: Significant difference between groups in UI but not AI. (UI: p = 0.03), (AI: p = 0.33) At 12 months: No significant difference between groups. Intervention group: UI, *n (%)* APFQ: Baseline: 41 (100) APFQ: Evaluation 6 months: 21 (57) APFQ: Evaluation 12 months: 28 (76) AI, *n (%)* APFQ: Baseline: 26 (63) APFQ: Evaluation 6 months: 21 (58) APFQ: Evaluation 12 months: 23 (60) Control group: UI, *n (%)* APFQ: Baseline: 43 (100) APFQ: Evaluation 6 months: 31 (82) APFQ: Evaluation 12 months: 34 (81) AI, *n (%)* APFQ: Baseline: 33 (77) APFQ: Evaluation 6 months: 27 (71) APFQ: Evaluation 12 months: 26 (62)
Von Bargen et al. 2021 [45] RCT USA	Women with grade 3–4 puerperal hernia. Mean age: 32.7 (2.7) Mean number of pregnancies:1.1 Number randomized: n = 50 Intervention: n = 25 Control: n = 25	Start: 2 weeks pp. Duration: 3 months. PMFT + feedback from physiotherapist Information about the function and anatomy of the pelvic floor. Weekly follow-up with a physiotherapist with verbal and tactile feedback on correct pelvic floor contraction. Individualized and progressive home training program by physiotherapist.	Standard care, not specified.	Evaluation: 3 months. UDI − 6 and Colorectal-anal Distress Inventory 8 (CRADI – 8) Primary outcome measure.	Significant difference between groups. (UI: p = 0.02), (AI: p = 0.01) Intervention group: UI, *Median (IQR)* UDI – 6: Baseline: 8.3 (0.0–16.7) UDI – 6: Evaluation: 0.0 (0.0–8.3) AI, *Median (IQR)* CRADI – 8: Baseline: 16.7 (4.2–33.3) CRADI – 8: Evaluation: 0.0 (0.0–8.3) Control group: UI, *Median (IQR)* UDI – 6: Baseline: 0.0 (0.0–10.4) UDI – 6: Evaluation: 0.0 (0.0–8.3) AI, *Median (IQR)* CRADI – 8: Baseline: 8.3 (0.0–22.9) CRADI – 8: Evaluation: 10.4 (0.0–20.8)
Wu et al. 2021 [46] CCT Taiwan	Women with grade 2 puerperal hernia. Mean age: 32.1 (4.9) Mean number of pregnancies: primiparous women only Number randomized: n = 75 Intervention: n = 38 Control: n = 37	Start: 6 weeks pp. Duration: 6 weeks. PMFT + feedback from biofeedback and physiotherapist Instructions through palpation, verbal, and written instructions on proper pelvic floor contraction. Daily home training program with biofeedback. Weekly follow-up with a physiotherapist on the phone, as well as two physical follow-ups.	Written instructions on proper pelvic floor contraction and advice on pelvic floor training.	Evaluation: 6 months. UDI − 6 Primary outcome measure.	No significant difference between groups. Intervention group: UI, *Mean (SD)* UDI – 6: Baseline: 3.2 (2.5) UDI – 6: Evaluation: 0.9 (1.7) Control group: UI, *Mean (SD)* UDI – 6: Baseline: 2.6 (2.0) UDI – 6: Evaluation: 1.3 (1.4)

Note: pp = post-partum; UI = urinary incontinence; AI = anal incontinence; RCT = randomized controlled trial: CCT = controlled clinical trial; ICIQ-UI SF = The International Consultation on Incontinence Questionnaire Urinary Incontinence Short Form; UDI – 6 = Urinary Distress Inventory Short Form; FISI = The Fecal Incontinence Severity Index; CCI = Cleveland Clinic Continence Score; APFQ = Australian Pelvic Floor Questionnaire; CRADI-8 = Colorectal-anal Distress Inventory 8; IQR = Interquartile Range

**Table 6:** Grading of methodological quality and probative value

Author	Ahlund et al. [39]	Hilde et al. [40]	Johannessen et al. [41]	Oakley et al. [42]	Perice et al. [43]	Sigurdardottir et al. [44]	Von Bargen et al. [45]	Wu et al. [46]
PEDro score	5	6	5	5	4	6	6	3
Methodological quality	Medium	High	Medium	Medium	Medium	High	High	Low
Probative value	Low	Medium	Low	Low	Low	High	Medium	Low

### Probative value

One of the studies was considered to have a high probative value [44]. Two studies were considered to have a medium level of evidence [40, 45], and five studies had a low level of evidence [39, 41–43, 46]. Two study was considered to have high methodological quality, but a medium level of evidence due to number of dropouts [45] or because the groups were not equal at baseline [40]. Four studies were considered to have medium methodological quality, but low evidentiary value [39, 41–43] due to not reporting or not homogeneous baseline values [42, 43], problem with the power calculation [39, 43] and an excessive number of dropouts [39, 41]. In addition, one study considered having low methodological quality was also classified as low level of evidence due to not perform a power calculation, lack of random allocation and high number dropouts [46] (See Table 5).

### Clinical relevance

Based on the assessment of clinical relevance, no study achieved a score of “yes” on all five of Furlan’s questions (see Table 7) [37]. Seven studies had sufficient reporting of population and relevant outcome measures to be clinically relevant [39–41, 44–46]. The interventions were described sufficiently by five studies [39–41, 44, 46]. The effect size could be calculated with Cohen’s d in two studies, which resulted in a low effect size [41, 46]. The other studies did not present applicable data that could be used to calculate clinical relevance, nor did these studies report any corresponding measure for clinical relevance [39, 40, 42–45]. No study reported a comparison between the benefit of the intervention with potential risks and costs. As five yes replies were required for a study to be considered clinically relevant, no study was assessed as clinically relevant.

**Table 7:** Assessment of clinical relevance according to Furlan [37]

Author	Ahlund et al. [39]	Hilde et al. [40]	Johannessen et al. [41]	Oakley et al. [42]	Perice et al. [43]	Sigurdardottir et al. [44]	Von Bargen et al. [45]	Wu et al. [46]
1.	Yes	Yes	Yes	Yes	Unclear	Yes	Yes	Yes
2.	Yes	Yes	Yes	Unclear	No	Yes	Unclear	Yes
3.	Yes	Yes	Yes	Yes	Unclear	Yes	Yes	Yes
4.	N/A	N/A	No d = 0.26 (St. Mark’s scores)	N/A	N/A	N/A	N/A	No d = 0.26 (UDI-6)
5.	No	No	No	No	Unclear	No	No	No

### Grading of evidence

Three studies reported significant between-group differences [41, 44, 45], with reduced incontinence symptoms observed among participants in the intervention groups. One of these was considered to have a high value of evidence [44], one was considered to have a medium value of evidence [45], and one was considered to have a low value of evidence [41]. Five studies did not report any significant difference between groups [39, 40, 42, 43, 46]. Of these, one study had a medium level of evidence [40], and four studies had a low level of evidence [39, 41, 43, 46]. There are not enough studies with a high level of evidence, and no study was assessed as clinically relevant. In addition, the results in the included studies are contradictory; the scientific basis is therefore considered to be insufficient.

## Discussion

This systematic review demonstrated that there is a lack of sufficient scientific evidence for the effect of PFMT with feedback from a physiotherapist and/or biofeedback for postpartum UI and AI.

The majority of the included studies in this systematic review had a medium to low level of evidence, which presents the risk of systematic error [32]. Five studies recruited the number of participants acquired to reach power [40–42, 44, 45]. However, four of the eight studies had a large participant dropout rate [39, 41, 45, 46]. An inadequate number of participants and high dropout rate leads to an increased risk of imprecision [32]. Dropouts could indicate difficulties adhering to the intervention; Pierce et al. [43] report that one of the reasons for low compliance is the time-consuming nature of the intervention.

In physiotherapy studies with active interventions, it is difficult to blind participants and therapists, which is the case in all reviewed studies. It has been suggested that lack of blinding increases the risk of treatment errors, because the expectations of the participant and the therapist can unintentionally affect the outcome. However, the effect of a lack of blinding in rehabilitation research is still inconclusive [50]. In one of the included studies, there is insufficient reported data and unclear reporting of baseline [43], which increases the risk of reporting errors [32]. This lowers the clinical relevance and transferability is reduced.

Despite the heterogeneity in the population and intervention groups in the included studies, the studies with significant between-group differences account for the heterogeneity [41, 44, 45]. This could indicate that women with different types of problems can benefit from feedback from a physiotherapist and/or biofeedback. Even so, the results are not specific, and none of the feedback types differed in terms of effect or non-effect. Results that are less specific are more difficult to apply clinically. Since the basis for physiotherapy is functional limitation and disability rather than medical diagnosis [51], the heterogeneity in included diagnosis might not be a limitation for the application of the results. On the other hand, it would have been beneficial to investigate UI and AI separately, as this likely would have provided a more consistent result. A Cochrane review, which evaluated the effect of pelvic floor training in adults with and without biofeedback on AI unrelated to childbirth, shows that the intervention has some scientific support [52]. The mechanisms that reduce symptoms in the general adult population may differ from the mechanisms in postpartum women, but the intervention nevertheless has scientific support. The included studies differ in how instructions for PFMT were provided in both intervention- and control groups, were some received verbal, some received written instructions, and some received both. To date, it is not clarified which type of instructions are most effective, and it has been suggested that there is no difference [53].

The included studies tended to evaluate the effect with varying time spans, which makes the results difficult to compare. One possible reason to lack of significant effects on incontinence symptoms postpartum could be time to follow up. If the time interval is too short between follow up points, the time interval may be too short to evaluate the effect of PMFT. Significant between-group differences were observed after three [45] and six [41, 45] months, respectively. Sigurdardottir et al. [44] reported that no differences between intervention and control groups could be observed regarding UI and AI after 12 months. This may indicate that PFMT with feedback from a physiotherapist and/or biofeedback accelerates natural healing in the first stage after delivery but does not produce a significant difference compared to the control group after one year [44]. The natural history tends to reduce incontinence symptoms in the first six months after childbirth [9]. The prevalence of UI has been shown to increase again after one year [9]. An overview by Mørkved et al. [54] reports that the research in the field is contradictory regarding the efficacy of pelvic floor training one year after childbirth. Furthermore, Mørkved et al. [54] conclude that the flattening of the effect may be due to the discontinuation of exercises among participants. A long-term effect cannot be expected if pelvic floor exercises are discontinuous [55]. Another factor leading to increased UI problems may be an increased demand on pelvic floor muscles through increased physical activity [56]. When the increase in abdominal pressure exceeds the threshold of the urinary sphincter, UI may occur. This applies to stress UI specifically [57].

Three studies showed statistically significant within-group differences in both the intervention and control groups [39, 41, 42]. Both groups performed PFMT, which may explain the within-group differences. The fact that all participants completed pelvic floor training [22] and underwent natural processes that reduce incontinence symptoms in the first six months after childbirth [9] can explain the occurrence of within group differences in both the intervention and control group, and thus explain why no significant between group differences were observed.

The instruments used were self-assessments, which are a measure of subjective experiences. Although all questionnaires were validated, there may be a discrepancy between patient-reported UI symptoms and objectively measured incontinence [58]. Such a discrepancy may relate to human support at physical appointments, where a relationship can be created between physiotherapist and study participant, which further increases the risk of bias, as neither participants nor therapists were blinded [59]. The self-estimate can also be influenced by internal factors, such as health status on the current day, and the individual’s subjective experience of symptoms and level of discomfort [59]. The results of the included studies would benefit from validation through other assessments [58].

### Application of the results

Together with the natural history [9] and continuous independent pelvic floor training, several of the participants in the included studies [39, 41, 42] experienced reduced discomfort within the first six months without receiving feedback from a physiotherapist and/or biofeedback. PFMT without any form of feedback may be suitable for patients who are confident practicing the exercises on their own and who find their symptoms less troublesome [21]. It can also be considered suitable for patients who prefer the flexibility of home exercises. Pelvic floor training with feedback can be particularly suitable for women who have difficulty performing a pelvic floor contraction correctly [60]. A lack of confidence in performing the PFMT properly or uncertainty about doing the right exercise are known barriers to compliance to PFMT [61, 62], and feedback could offer support to overcome that barrier. Feedback, from a physiotherapist and/or biofeedback, is important for successful pelvic floor therapy, since it can provide confirmation that the patient is correctly contracting muscles and ensure that the dosage of exercise is adapted to the individual [24]. It was not an inclusion criterion in this study, and in none of the included studies, that the women in the included studies could perform a correct pelvic floor contraction from start, which reflect the studied population, where difficulties to correctly contract is common [60]. Patients may also see a positive effect by spending more time with healthcare professionals [24]. PFMT has been found to be effective when supervised training is conducted [54]. Cochrane suggests that, in certain groups of women, it is possible that the effects of PFMT would be greater with targeted treatment approaches [21]. Physiotherapists should therefore continue to provide individualised treatment.

A systematic review [61] was performed that focused on prenatal and postnatal PFMT in prevention and treatment for pelvic organ prolapse and other pelvic floor dysfunction. The results showed a positive effect of pelvic-floor muscle training in prepartum and postpartum periods on pelvic-floor dysfunction prevention, particularly in UI symptoms. Based on their results, the authors argue for that there should be national strategies for pregnancy and postpartum rehabilitation programs due to a high prevalence of pelvic floor dysfunction in the general female population. In relation to that study, the present study further emphasises the importance of tailoring PFMT to suit the needs of the individual.

### Future studies

There is a great need for additional high-quality randomised controlled trials that evaluate the effect of pelvic floor muscle training with different kinds of feedback on UI and AI separately [21, 31]. Considering the natural history and increased UI symptoms one year after childbirth, there is also a need for a longer follow-up period. Future studies could benefit from supplementing the evaluation with objective measures, such as the Pad Test provocation test [62]. Studies comparing feedback from a physiotherapist with biofeedback will be necessary to further optimise PFMT in post-partum women. To date, there is, as far as the authors know, no studies making such comparison.

### Strengths and limitations

A strength of the current review is its systematic performance according to guidelines [32]. The three databases used hold a wide range of medical research. As maternity care overlaps several research fields, the databases were selected to obtain a large range of literature. In terms of methodology, the extensive search strategy was a strength, as it ensures that available research was found. To reduce the risk of bias, the selection process and all assessments were done by at least two authors independently [32]. The standardised PEDro scale was used in the assessment of methodological quality as an additional factor to reduce subjectivity [32].

This systematic review has limitations regarding the literature search method and selection criteria. The study aims to focus on the role of physiotherapists in the maternity care. It may increase the risk of bias, as the research question and selection criteria could have resulted in excluding relevant studies. The focus was chosen to highlight the competence of physiotherapists in the field. Despite this, the interventions can be used by several skilled professionals depending on national guidelines. The collection of literature was done in English only, as the authors did not have the resources to obtain text from other languages and guarantee a correct translation and interpretation. The language limitations could increase the risk of systematic bias and the risk of excluding relevant studies [63]. Studies published earlier than 2012 were excluded. A wider time span could result in a larger base and thus enable a narrower question and a more specific result. To reduce the risk of comparing different forms of technological equipment and to conduct an updated conclusion based on recent research, the inclusion criteria was chosen [64].

A limitation of the current systematic review is the use of the Britton model of probative value and grading of evidence, which is not internationally established [36]. The Britton model aims to assess the trustworthiness of an individual study’s conclusions and to generate a compilation of all conclusions. Despite the fact that the Britton model is not an established model, it considers several aspects and aims to identify systematic limitations. The current study aims to describe the method transparently.

Another limitation is the width and variation in population, intervention, control, and assessment measures, which generates results that are less specific. This also rules out the performance of a meta-analysis. The wide inclusion criteria was required to gather a sufficiently large base, since there are few randomised controlled studies in the area. However, the broad population of women with and without injury may reflect the patient group encountered in the clinic, as there is a large number of undetected injuries [65]. It is not possible to draw specific conclusions about the effect of the intervention on a specific population. However, with a focus on functional limitations and disability rather than medical diagnosis [51], the broad population could be considered adequate.

## Conclusion

There is insufficient scientific evidence for the effect of PFMT with feedback from a physiotherapist or biofeedback in postpartum incontinence compared to pelvic floor training recommendations alone. Based on the results of this systematic review, it is not possible to draw new conclusions about the evidence for pelvic floor training with feedback. In individualised treatment, the use of feedback from a physiotherapist could fill a need for certain patients. Additional high-quality studies are needed to draw scientifically based conclusions about this treatment option for postpartum incontinence.

### Electronic supplementary material

Below is the link to the electronic supplementary material.

Supplementary Material 1

Supplementary Material 2

Supplementary Material 3

## Data Availability

All data generated or analysed during this study are included in this published article and its supplementary information files, together with all included full texts (no 39–46 in the reference list).

## List of abbreviations

UI: urinary incontinence
AI: anal incontinence
PFMT: pelvic floor muscle training
RCT: randomised controlled trial
CCT: controlled clinical trial
ICIQ-UI SF: The international consultation on incontinence questionnaire urinary incontinence short form
UDI – 6: Urinary distress inventory short form
FISI: The fecal incontinence severity index
APFQ: Australian pelvic floor questionnaire
CRADI-8: Colorectal-anal distress inventory 8
N/A: not applicable
