# Supplementary material for: Pelvic floor muscle training with biofeedback or feedback from a physiotherapist for urinary and anal incontinence after childbirth - a systematic review
Source: BMC Womens Health. 2023 Nov 18;23:618. doi: 10.1186/s12905-023-02765-7 (PMC10657595; doi:10.1186/s12905-023-02765-7)
Supplement: Supplementary file 1 — Supplementary Material 1 [file 12905_2023_2765_MOESM1_ESM.docx]

Additional file 3

Grading of methodological quality according to the PEDro scale

| Author | Ahlund et al. 2013 | Hilde et al.  2013 | Johannessen et al.  2017 | Oakley et al.  2016 | Peirce et al.  2013 | Sigurdardottir  et al.  2020 | Von Bargen et al.  2021 | Wu et al.  2021 |
| --- | --- | --- | --- | --- | --- | --- | --- | --- |
| 1. Eligibility criteria were specified * | Yes | Yes | Yes | Yes | Yes | Yes | Yes | No |
| 2. Subjects were randomly allocated to groups (in a crossover study, subjects were randomly allocated an order in which treatments were received) | Yes | Yes | Yes | Yes | Yes | Yes | Yes | No |
| 3. Allocation was concealed | Yes | Yes | No | Yes | Yes | Yes | Yes | No |
| 4. The groups were similar at baseline regarding the most important prognostic indicators | Yes | No | Yes | No | No | Yes | Yes | Yes |
| 5. There was blinding of all subjects | No | No | No | No | No | No | No | No |
| 6. There was blinding of all therapists who  administered the therapy | No | No | No | No | No | No | No | No |
| 7. There was blinding of all assessors who measured at least one key outcome | No | No | No | No | No | No | No | No |
| 8. Measures of at least one key outcome were obtained from more than 85% of the subjects initially allocated to groups | No | Yes | No | Yes | Yes | Yes | No | No |
| 9. All subjects for whom outcome measures were  available received the treatment or control condition as allocated or, where this was not the case, data for at least one key outcome was analysed by “intention to  treat”. | No | Yes | Yes | No | No | No | Yes | No |
| 10. The results of between-group statistical  comparisons are reported for at least one key outcome. | Yes | Yes | Yes | Yes | Yes | Yes | Yes | Yes |
| 11. The study provides both point measures and measures of variability for at least one key outcome. | Yes | Yes | Yes | Yes | No | Yes | Yes | No |
| Total score (max = 10) | 5 | 6 | 5 | 5 | 4 | 6 | 6 | 3 |

**Not included in the total score*
