# Supplementary material for: Pelvic floor muscle training with biofeedback or feedback from a physiotherapist for urinary and anal incontinence after childbirth - a systematic review
Source: BMC Womens Health. 2023 Nov 18;23:618. doi: 10.1186/s12905-023-02765-7 (PMC10657595; doi:10.1186/s12905-023-02765-7)
Supplement: Supplementary file 2 — Supplementary Material 2 [file 12905_2023_2765_MOESM2_ESM.docx]

**Additional file 2.** List of articles not included after fulltext reading

1. Kolberg Tennfjord M, Hilde G, Staer-Jensen J, Siafarikas F, Engh ME, Bø K. Effect of postpartum pelvic floor muscle training on vaginal symptoms and sexual dysfunction-secondary analysis of a randomised trial. BJOG. 2016 Mar;123(4):634-42.

- *Wrong population*

1. Lange S, Tabibi E, Naumann G, Lange R. Comparison of pessaries to pelvic floor training to treat postpartum urinary incontinence: results from a randomized pragmatic trial (brest). AUGS. 2022 Jun;28(6):26-7.

- *Not avaliable in fulltext*

1. Lio Z, Sun Z. Effects of electromyographic (EMG) biofeedback-guided pelvic floor muscle training on postpartum stress urinary incontinence. Int J Clin Exp Med. 2019;12(4):3742-49.

- *Wrong population*

1. Movahedi M, Torabipoor MS, Mohammadi MS, Shariat M, Haghollahi F, Hajihashem M. The effect of postpartum physiotherapy on sexual function and incontinence of primparous women in Al-Zahra and shahid Beheshti hospitals of Isfahan: a randomized clinical trial.TUMS. 2021;79(5):351‐60.

- *Not avaliable in fulltext*

1. O'Herlihy C. Early home feedback physiotherapy compared with pelvic floor exercises soon after third degree perineal tear: a randomised trial. IUGA. 2012 Aug;03(23):107.

- *Not avaliable in fulltext*

1. Shivkumar R, Srivastava N, Gupta J. Effects of Bladder Training and Pelvic Floor Muscle Exercise in Urinary Stress Incontinence During Postpartum Period. IJPOT. 2015 Oct;9(4):194–8.

- *Wrong intervention*

1. Von Bargen E, Haviland MJ, Chang OH, McKinney JL, Hacker MR, Elkadry EA. Evaluation of postpartum pelvic floor physical therapy on obstetrical anal sphincter injury. AUGS. 2018 Sep;24(5):18-55.

- *Not avaliable in fulltext*

1. Wen X‐H, Shi S‐Q, Wang J‐Y. Pelvic muscles exercise for postpartum stress urinary incontinence. China practical medicine. 2010;5(15):72‐3.

- *Not avaliable in fulltext*
