# Supplementary material for: Pelvic floor muscle training with biofeedback or feedback from a physiotherapist for urinary and anal incontinence after childbirth - a systematic review
Source: BMC Womens Health. 2023 Nov 18;23:618. doi: 10.1186/s12905-023-02765-7 (PMC10657595; doi:10.1186/s12905-023-02765-7)
Supplement: Supplementary file 3 — Supplementary Material 3 [file 12905_2023_2765_MOESM3_ESM.docx]

**Additional file 1**

Literature search in databases

| Database  and date |  | Search block | Number of hits |
| --- | --- | --- | --- |
| PubMed  2022-09-30 | #1 | ((perineal tear OR sphincter injury OR sphincter trauma OR episiotomy OR birth trauma OR perineotomy OR perineal laceration OR perineal trauma OR perineal injury OR obstetric damage OR sphincter damage OR obstetric rupture OR obstetric injury OR anal sphincter tear OR third degree tear OR fourth degree tear OR OASIS OR anal sphincter damage OR obstetric anal sphincter laceration OR obstetric anal sphincter injury OR obstetric anal sphincter rupture OR anal incontinence OR fecal incontinence  OR urinary incontinence OR Second-degree perineal laceration) | 51.880 |
|  | #2 | (pelvic floor OR anal sphincter muscles OR pelvic floor muscles) | 9.705 |
|  | #3 | (Exercise OR muscle contraction OR physiotherapy OR pfmt OR muscle training OR training OR Rehabilitation OR Therapeutic Exercise  OR motor activity OR biofeedback OR EMG OR electromyography OR Kegel exercise OR muscles OR muscle exercises OR supervised OR supervised pelvic floor muscle training) | 2.669.175 |
|  | #4 | (postpartum) | 53.944 |
|  | #5 | (randomized controlled trial OR randomized controlled trial OR randomized OR randomized OR controlled OR trial OR RCT) | 1.431.020 |
|  | #6 | #1 AND #2 AND #3 AND #4 AND #5 | 102 |

| Database  and date |  | Search block | Number of hits |
| --- | --- | --- | --- |
| PubMed  2023-10-12 | #1 | ((perineal tear OR sphincter injury OR sphincter trauma OR episiotomy OR birth trauma OR perineotomy OR perineal laceration OR perineal trauma OR perineal injury OR obstetric damage OR sphincter damage OR obstetric rupture OR obstetric injury OR anal sphincter tear OR third degree tear OR fourth degree tear OR OASIS OR anal sphincter damage OR obstetric anal sphincter laceration OR obstetric anal sphincter injury OR obstetric anal sphincter rupture OR anal incontinence OR fecal incontinence  OR urinary incontinence OR Second-degree perineal laceration) | 58.599 |
|  | #2 | (pelvic floor OR anal sphincter muscles OR pelvic floor muscles) | 11.160 |
|  | #3 | (Exercise OR muscle contraction OR physiotherapy OR pfmt OR muscle training OR training OR Rehabilitation OR Therapeutic Exercise  OR motor activity OR biofeedback OR EMG OR electromyography OR Kegel exercise OR muscles OR muscle exercises OR supervised OR supervised pelvic floor muscle training) OR physical therapy | 3.077.866 |
|  | #4 | (postpartum) | 61.738 |
|  | #5 | (randomized controlled trial OR randomized controlled trial OR randomised OR randomised OR controlled OR trial OR RCT) | 1.611.649 |
|  | #6 | #1 AND #2 AND #3 AND #4 AND #5 | 124 |

| and date |  | Search block | Number of hits |
| --- | --- | --- | --- |
| Cochrane  2022-09-30 | #1 | ((perineal tear OR sphincter injury OR sphincter trauma OR episiotomy OR birth trauma OR perineotomy OR perineal laceration OR perineal trauma OR perineal injury OR obstetric damage OR sphincter damage OR obstetric rupture OR obstetric injury OR anal sphincter tear OR third degree tear OR fourth degree tear OR OASIS OR anal sphincter damage OR obstetric anal sphincter laceration OR obstetric anal sphincter injury OR obstetric anal sphincter rupture OR anal incontinence OR fecal incontinence  OR urinary incontinence OR Second-degree perineal laceration) | 11.016 |
|  | #2 | (pelvic floor OR anal sphincter muscles OR pelvic floor muscles) | 3.778 |
|  | #3 | (Exercise OR muscle contraction OR physiotherapy OR pfmt OR muscle training OR training OR Rehabilitation OR Therapeutic Exercise  OR motor activity OR biofeedback OR EMG OR electromyography OR Kegel exercise OR muscles OR muscle exercises OR supervised OR supervised pelvic floor muscle training) | 199.031 |
|  | #4 | (postpartum) | 10.218 |
|  | #5 | (randomized controlled trial OR randomized controlled trial OR randomized OR randomized OR controlled OR trial OR RCT) | 1.282.034 |
|  | #6 | #1 AND #2 AND #3 AND #4 AND #5 | 220 |

| Database  and date |  | Search block | Number of hits |
| --- | --- | --- | --- |
| Cochrane  2023-10-12 | #1 | ((perineal tear OR sphincter injury OR sphincter trauma OR episiotomy OR birth trauma OR perineotomy OR perineal laceration OR perineal trauma OR perineal injury OR obstetric damage OR sphincter damage OR obstetric rupture OR obstetric injury OR anal sphincter tear OR third degree tear OR fourth degree tear OR OASIS OR anal sphincter damage OR obstetric anal sphincter laceration OR obstetric anal sphincter injury OR obstetric anal sphincter rupture OR anal incontinence OR fecal incontinence  OR urinary incontinence OR Second-degree perineal laceration) | 11.588 |
|  | #2 | (pelvic floor OR anal sphincter muscles OR pelvic floor muscles) | 4.321 |
|  | #3 | (Exercise OR muscle contraction OR physiotherapy OR pfmt OR muscle training OR training OR Rehabilitation OR Therapeutic Exercise  OR motor activity OR biofeedback OR EMG OR electromyography OR Kegel exercise OR muscles OR muscle exercises OR supervised OR supervised pelvic floor muscle training OR physical therapy) | 251.415 |
|  | #4 | (postpartum) | 11.755 |
|  | #5 | (randomized controlled trial OR randomized controlled trial OR randomised OR randomised OR controlled OR trial OR RCT) | 1.416.901 |
|  | #6 | #1 AND #2 AND #3 AND #4 AND #5 | 258 |

| and date |  | Search block | Number of hits |
| --- | --- | --- | --- |
| CINAHL  2022-09-30 | #1 | ((perineal tear OR sphincter injury OR sphincter trauma OR episiotomy OR birth trauma OR perineotomy OR perineal laceration OR perineal trauma OR perineal injury OR obstetric damage OR sphincter damage OR obstetric rupture OR obstetric injury OR anal sphincter tear OR third degree tear OR fourth degree tear OR OASIS OR anal sphincter damage OR obstetric anal sphincter laceration OR obstetric anal sphincter injury OR obstetric anal sphincter rupture OR anal incontinence OR fecal incontinence  OR urinary incontinence OR Second-degree perineal laceration) | 4.479 |
|  | #2 | (pelvic floor OR anal sphincter muscles OR pelvic floor muscles) | 1.101 |
|  | #3 | (Exercise OR muscle contraction OR physiotherapy OR pfmt OR muscle training OR training OR Rehabilitation OR Therapeutic Exercise  OR motor activity OR biofeedback OR EMG OR electromyography OR Kegel exercise OR muscles OR muscle exercises OR supervised OR supervised pelvic floor muscle training) | 125.079 |
|  | #4 | (postpartum) | 6.066 |
|  | #5 | (randomized controlled trial OR randomized controlled trial OR randomised OR randomised OR controlled OR trial OR RCT) | 102.239 |
|  | #6 | #1 AND #2 AND #3 AND #4 AND #5 | 7 |

| Database  and date |  | Search block | Number of hits |
| --- | --- | --- | --- |
| CINAHL  2023-10-12 | #1 | ((perineal tear OR sphincter injury OR sphincter trauma OR episiotomy OR birth trauma OR perineotomy OR perineal laceration OR perineal trauma OR perineal injury OR obstetric damage OR sphincter damage OR obstetric rupture OR obstetric injury OR anal sphincter tear OR third degree tear OR fourth degree tear OR OASIS OR anal sphincter damage OR obstetric anal sphincter laceration OR obstetric anal sphincter injury OR obstetric anal sphincter rupture OR anal incontinence OR fecal incontinence  OR urinary incontinence OR Second-degree perineal laceration) | 4.606 |
|  | #2 | (pelvic floor OR anal sphincter muscles OR pelvic floor muscles) | 1.158 |
|  | #3 | (Exercise OR muscle contraction OR physiotherapy OR pfmt OR muscle training OR training OR Rehabilitation OR Therapeutic Exercise  OR motor activity OR biofeedback OR EMG OR electromyography OR Kegel exercise OR muscles OR muscle exercises OR supervised OR supervised pelvic floor muscle training OR physical therapy) | 141.601 |
|  | #4 | (postpartum) | 6.501 |
|  | #5 | (randomized controlled trial OR randomized controlled trial OR randomized OR randomized OR controlled OR trial OR RCT) | 108.981 |
|  | #6 | #1 AND #2 AND #3 AND #4 AND #5 | 7 |

 The primary search was performed in September 2022. In October 2023 a complementary search was performed, including publications up to present date, and also adding the search term ‘physical therapy’ to block #3 in all search engines.
